# Crystal structure of TRAF1 TRAF domain and its implications in the TRAF1-mediated intracellular signaling pathway

**DOI:** 10.1038/srep25526

**Published:** 2016-05-06

**Authors:** Chang Min Kim, Jae Young Choi, Eijaz Ahmed Bhat, Jae-Hee Jeong, Young-Jin Son, Sunghwan Kim, Hyun Ho Park

**Affiliations:** 1 Department of Biochemistry, Yeungnam University, Gyeongsan, 712-749, South Korea; 2 Pohang Accelerator Laboratory, Pohang University of Science and Technology, Pohang, 790-784, South Korea; 3 New Drug Development Center, Daegu-Gyungpook Medical Innovation Foundation, Daegu, 701-310, South Korea

## Abstract

TNF-receptor associated factor (TRAF) proteins are key adaptor molecules containing E3 ubiquitin ligase activity that play a critical role in immune cell signaling. TRAF1 is a unique family of TRAF lacking the N-terminal RING finger domain. TRAF1 is an important scaffold protein that participates in TNFR2 signaling in T cells as a negative or positive regulator via direct interaction with TRAF2, which has recently been identified as a pro-apoptotic regulator in neuronal cell death. Here, we report the first crystal structure of the TRAF1 TRAF domain containing both the TRAF-N coiled-coil domain and the TRAF-C domain. Our structure reveals both similarities and differences with other TRAF family members, which may be functionally relevant to TRAFs. We also found that the TRAF-N coiled-coil domain of TRAF1 is critical for the trimer formation and stability of the protein. Finally, we found that conserved surface residues on the TRAF1 TRAF domain that might be binding hot spots that are critical for interaction with signaling molecules.

TNF-receptor associated factor (TRAF) proteins are key intracellular signaling molecules in the tumor necrosis receptor (TNFR) and toll like receptor (TLR) family signaling pathways that play critical roles in the immune system^1^,^2^,^3^. TRAF proteins function as scaffolds that mediate the interactions between members of the TNF receptor (TNF-R) family and downstream effector molecules, which are primarily protein kinases, including IRAKs, RIP1, RIP2, TAK1, MEKK1, and ASK1^4^,^5^,^6^,^7^. Several ubiquitin ligases are also recruited to membrane receptors via direct interaction with TRAFs^8^. Antagonistic roles of TRAFs in the TNFR and TLR signaling pathways have also been reported^9^,^10^. Most TRAFs contain an N-terminal RING finger domain^11^, which is found in many E3 ubiquitin ligases comprising the core of the ubiquitin ligase catalytic domain. Based on this, the E3 ligase activity of TRAFs has also been reported^12^. Therefore, TRAFs function as both adaptor and E3 ubiquitin ligases in immune cell signaling pathways. Because of their involvement in many human diseases, including cancer, autoimmunity, and inflammatory diseases, TRAFs have been suggested as suitable targets for therapeutic intervention^13^.

Seven TRAF proteins, TRAF1–TRAF7, have been identified in mammals^2^, all of which except TRAF7 contain the C-terminal homology domain of ~230 amino acids known as the TRAF domain. The TRAF domain, which is composed of 7–8 anti-parallel β-strand folds followed by a coiled-coil region that mediates protein interactions, usually forms mushroom-like trimeric structures in solution^14^. Despite the structural similarity of the TRAF domain, each TRAF exhibits specific biological functions possessing specificity to interacting upstream receptors and downstream effector molecules.

TRAF1 is a well-known adaptor molecule that regulates the activation of NF-kappaB and JNK^15^,^16^. TRAF1 was first identified as TNF receptor type 2 (TNFR2) binding protein^17^. Although the cellular functions of TRAF1 are not well understood when compared with those of other TRAF family members, several studies have indicated that TRAF1 participates in TNFR2 signaling in T cells as a negative regulator via direct interaction with TRAF2^17^. However, recent studies described a positive regulatory role for TRAF1 downstream of TNFR2, GITR, 4-1BB, LMP1 and CD30 and in association with TRAF2^18^,^19^,^20^.

One of the main functions of TRAF1 is suppression of TNF-α or T cell receptor (TCR)-mediated apoptosis, which is known to be an anti-apoptotic function of TRAF1^21^. However, recent studies have shown pro-apoptotic function of TRAF1 in neuronal cell death, offering a novel therapeutic target for stroke treatment with a relatively longer timeframe of use^22^. TRAF1 is considered a unique family among TRAFs in that it lacks the N-terminal RING finger domain that is critical for relaying the signal to downstream effectors. These findings indicate that the working mechanism of TRAF1 might be different from other TRAF proteins.

Because of the importance of TRAF proteins in signal transductions, all structural information regarding the TRAF domain of TRAF family, except the TRAF1 TRAF domain, have been elucidated. Despite the emerging roles of TRAF1 in human disease states, including recently reported hepatic and cerebral ischemia/reperfusion injury^22^,^23^ and anaplastic large cell lymphoma^24^, the structure of TRAF1 has not been determined to date. Here, we report the first crystal structure of the TRAF1 TRAF domain at a resolution of 2.8 Å. Although the TRAF1 TRAF domain has the typical TRAF domain fold, our structure reveals both similarities and differences, which may be functionally relevant to TRAFs. We also found that the TRAF-N coiled-coil domain of TRAF1 is critical to the trimer formation and stability of the protein. Finally, further structure and sequence analysis revealed conserved surface residues on the TRAF1 TRAF domain that might be critical for interaction with signaling molecules.

## Results and Discussion

### Structure of the TRAF1 TRAF domain

**Figure 1:**
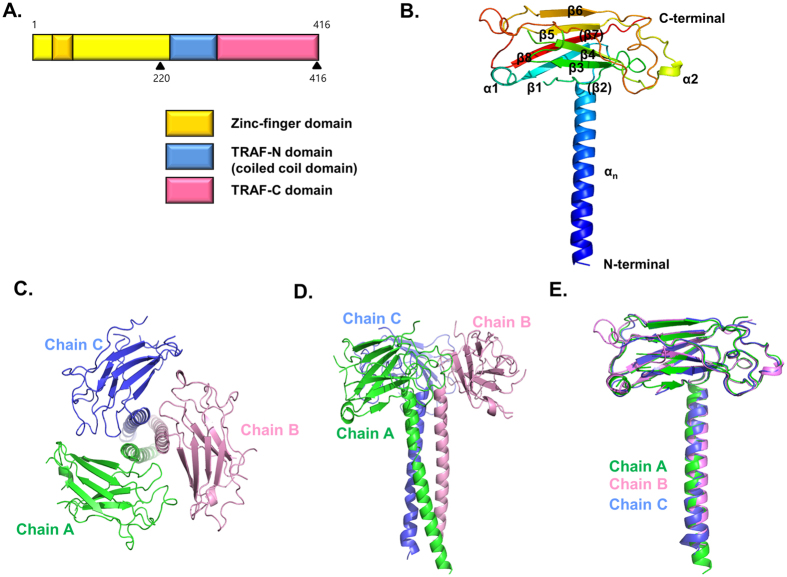
Crystal structure of the TRAF1 TRAF domain with the coiled-coil domain. (**A**) Domain boundary of TRAF1. The TRAF1 construct containing residues 220–416 used for the current structural study is indicated by black triangles. (**B**) Cartoon of the monomeric TRAF1 TRAF domain. The chain from the N- to C-termini is colored blue to red. Secondary structures including helices and sheets are labeled. (**C**,**D**) Cartoon of the trimeric TRAF1 TRAF domain. Chains (**A**–**C**) are shown separately in different colors. The top view (**C**) and side view (**D**) are shown. (**E**). Superposition of the structure of each chain.

Seven TRAF proteins have been identified in mammals, TRAF1–TRAF7, all of which except TRAF7 contain the TRAF domain. TRAF1 is considered a unique family of TRAFs in that it does not contain the N-terminal RING finger domain, which is another common feature in the TRAF family that may be critical for the E3 ubiquitin ligase function (Fig. 1A). The TRAF domain, containing both the TRAF-N coiled-coil domain and TRAF-C domain, is located at the C-terminus of TRAF1 as in other TRAF family members.

The 2.8 Å crystal structure of the TRAF domain (corresponding to amino acids 220–416) consisting of the TRAF-N coiled-coil domain and the TRAF-C anti-parallel β-sandwich domain was solved using the molecular replacement (MR) method and refined to Rwork = 21.0% and Rfree = 26.2%. The crystallographic and refinement statistics are summarized at Table 1. The previously solved human TRAF3 structure (PDB id: 4GHU), which has 49% amino-acid sequence homology with that of the TRAF1 TRAF domain, was used as the search model^25^. The structure of the TRAF1 TRAF domain revealed that TRAF-C comprises two α-helices (α1–α2) and six β-sheets (β1–β8). β2 and β7, which have been shown at other TRAF structures, were not detected and were replaced by the loop structure in the TRAF1 structure (Fig. 1B). TRAF-N coiled-coil domain (α_n_) was detected at the N-terminus. The overall fold of the TRAF1 TRAF domain was similar to that of other TRAF domains. Specifically, they resembled the shape of a mushroom, in which the TRAF-N coiled-coil domain forms the stalk and the TRAF-C domain forms the cap.

There was one trimer (three monomers) in the asymmetric unit, chain A, chain B, and chain C, and three molecules related by near perfect three-fold symmetry (Fig. 1C,D). A model of chain A was constructed from residues 223 to 416, while those of chain B and chain C were built from residues 228 to 416 and from residues 227 to 416, respectively. The six anti-parallel β-sheets comprising residues 268–273, 305–308, 325–328, 345–350, 358–362, and 406–412 were numbered β1, β3, β4, β5, β6, and β8, respectively (Fig. 1B). The probable β-sheets, which were detected at other TRAFs and comprised residues 292–297 and 379–386, are indicated as (β2) and (β7), respectively. Several loops including residues 282–287, 314–320, and 390–397 for chain A, 283–287 and 317–319 for chain B, and 281–288, 314–321, and 392–398 for chain C, were not built in the model because of poor electron density. The structures of the three chains are nearly identical, having a root-mean-square deviation (R.M.S.D.) of 1.06 between the A and B chain, 1.26 between the B and C chain, and 1.62 between the A and C chain (Fig. 1E). The stalk consists of five coiled-coil heptad repeats. With our current TRAF1 structure, the TRAF domain structures of the entire TRAF family (TRAF1–TRAF6) were elucidated.

### Trimeric interface of the structure at the TRAF1 TRAF domain

**Figure 2:**
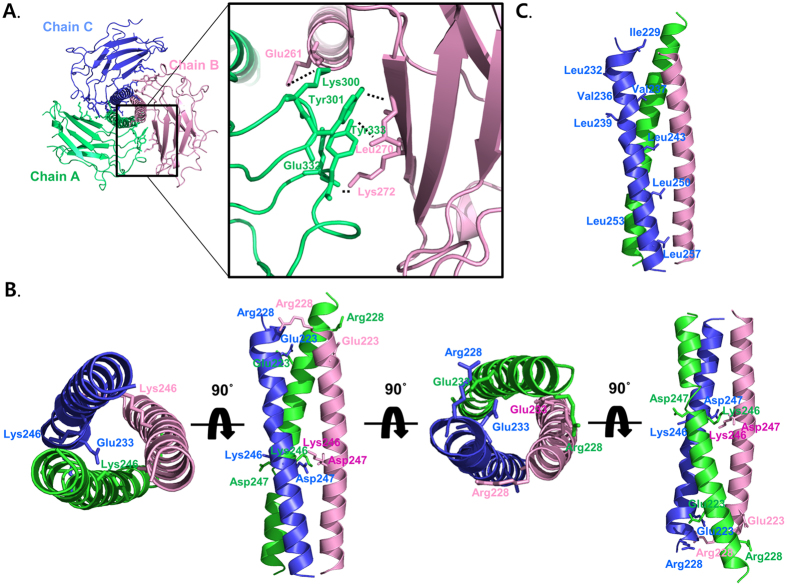
Trimeric interface of the structure at the TRAF1 TRAF domain. (**A**) The trimeric structure of the TRAF1 TRAF-C domain. Close-up of the interacting residues in the interface between two monomeric TRAF1 TRAF-C domains (green for chain (**A**) and pink for chain (**B**) is shown in the right panel. Residues involved in the contact are shown. (**A**) salt bridge formed between Glu332 from one chain and Lys272 from its counterpart is shown as a red dashed-line. All hydrogen bonds are shown as black dashed-lines. (**B**) The trimeric structure of the TRAF1 TRAF-N coiled-coil domain. Residues involved in the formation of two charged patches are shown. (**C**) The residues involved in the hydrophobic interactions with other coiled-coil chains are shown.

The structure of the trimeric TRAF1 TRAF domain exhibited a typical mushroom shape, in which the TRAF domain forms the cap and the coiled-coil domain forms the stalk. The trimeric TRAF-C domains were loosely packed with the narrow portion of the binding interface, while the TRAF-N coiled-coil domain was tightly packed with massive hydrophobic interactions and salt bridges. The trimeric interface of the TRAF1 TARF-C domain is formed by packing one end of the (β2)-β3 connecting loop, the β4-β5 connecting loop, against β1 of the neighboring monomer (Fig. 2A). The major force for formation of the trimeric interfaces of the TRAF1 TRAF-C domain was several hydrogen bonds formed between Glu261, Leu270 and Lys272 from one TRAF molecule (chain B) and Lys300, Tyr301, Glu332 and Tyr333 from the second molecule (chain A) (Fig. 2A). The trimeric interface of the TRAF1 TRAF-N coiled-coil domain is primarily formed by several hydrophobic patches and two salt bridge clusters (Fig. 2B). Hydrophobic patches are formed by Ile229, Leu232, Val236, Val237, Leu239, Leu243, Leu250, Leu253 and Leu257 from each chain (Fig. 2C). Two salt bridge clusters were formed at the beginning part, with Glu223 and Arg228 from each chain and the middle part with Lys246 and Asp247 of the TRAF1 TRAF-N coiled-coil domain. Because the massive interactions between inter-chains were located at the coiled-coil stalk region, the TRAF1 TRAF-N coiled-coil domain could be important for stabilization of trimer formation. The total surface areas formed by the three molecules were around 34,218 Å^2^ and 3264 Å^2^ and were buried upon complex formation, which corresponds to an average of 1088 Å^2^ per molecule.

### The function of the TRAF-N coiled-coil domain for the trimerization and the stability of the TRAF1 TRAF domain

**Figure 3:**
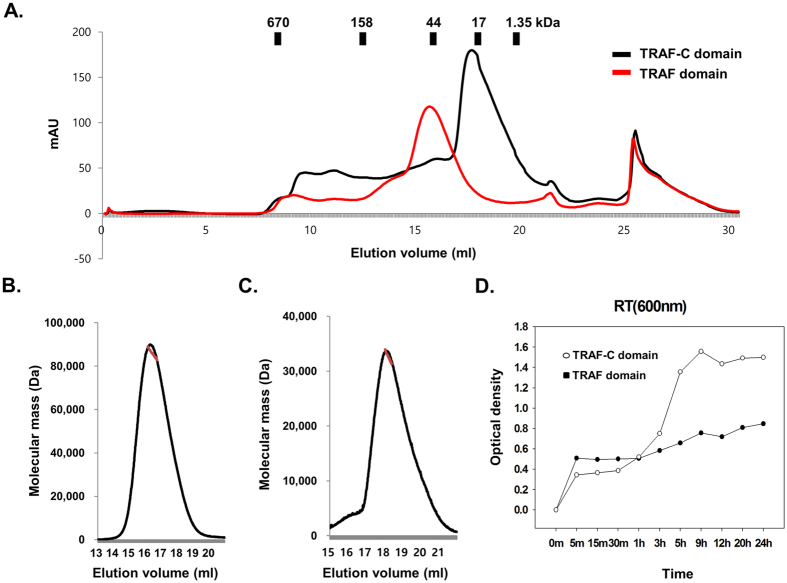
The function of the TRAF-N coiled-coil domain for the trimerization and the stability of the TRAF1 TRAF domain. (**A**) Comparison of gel-filtration chromatography profile between the TRAF1 TRAF domain containing the TRAF-N and TRAF-C domain and the TRAF1 TRAF-C domain. (**B**,**C**). Multi-angle light scattering (MALS) result for TRAF1 TRAF domain (**B**) and TRAF1 TRAF-C domain. The red line indicates the experimental molecular weight. (**D**) Comparison of solubility between the TRAF1 TRAF domain and the TRAF1 TRAF-C domain as a function of time. Both protein samples were at 1 mg/ml in 20 mM sodium citrate at pH 5.0 and 1 M NaCl. Turbidity of each sample at the indicated incubation time was measured using the optical density at 600 nm. Values are the means ± SD (n = 3).

The TRAF1 TRAF domain, which contains both the TRAF-N coiled-coil domain and TRAF-C domain and was used for the current structural study, was over-expressed in bacteria and purified by affinity chromatography followed by size-exclusion chromatography. Upon size-exclusion chromatography, the TRAF1 TRAF domain was eluted at around 16 ml, indicating that it forms a stable trimer in solution similar with other TRAF family members (Fig. 3A). Interestingly, we found that the TRAF1 TRAF-C domain (residues 253 to 416), which lacks the TRAF1 TRAF-N coiled-coil domain, failed to form a trimer in solution based on the size-exclusion chromatography (Fig. 3A). The stoichiometry of the TRAF1 TRAF domain and the TRAF1 TRAF-C domain were accurately confirmed by multi-angle light scattering (MALS). The calculated molecular weights of the monomeric TRAF1 TRAF domain (residues 220 to 416) and TARF1 TRAF-C domain (residues 253 to 416), including the C-terminal His-tag, were 29.4 kDa and 22.7 kDa, respectively, and the experimental molecular weights from MALS were 82.8 kDa (0.8% fitting error) and 31.5 kDa, respectively (Fig. 3B,C). Analysis by size-exclusion chromatography and MALS indicated that the TRAF1 TRAF domain exists as a trimer in solution, while the TRAF1 TRAF-C domain without the TRAF-N coiled-coil domain exists as a monomer in solution, indicating that the TRAF-N coiled-coil domain is critical for trimer formation of TRAF1. The loosely packed TRAF-C domain and tightly packed TRAF-N domain of TRAF-1 detected by the current structure explained why the TRAF-N coiled-coil domain is critical to the trimer formation of TRAF1 in solution.

Unlike other TRAFs, the TRAF1 TRAF domain was insoluble under physiological conditions, 20 mM Tris-HCl pH 8.0 and 150 mM NaCl. To make the TRAF1 TRAF domain soluble, we attempted solubility tests using different pH values and salt concentrations. We were finally able to solubilize TRAF1 in buffer containing 20 mM sodium citrate and 1 M NaCl, which is an extremely low pH and high salt level. During the solubility test, we realized that the TRAF1 TRAF-C domain alone precipitated readily about 1 hour after size-exclusion chromatography. Due to the insoluble nature of the TRAF-C domain, the solubility difference between the TRAF domain and TRAF-C domain was analyzed by different time points (Fig. 3D). Our analysis showed that the monomeric TRAF TRAF-C domain is much more insoluble than the with TRAF1 TRAF domain, which contains both the TRAF-N coiled-coil domain and the TRAF-C domain. These findings indicate that the TRAF-N coiled-coil domain is critical for trimer formation, and this trimerization is important to the solubility of TRAF1. Taken together, these findings indicate that the TRAF-N coiled-coil domain is critical for trimer formation, which is the functional unit of TRAF1. In addition, we showed that proper trimer formation of the TRAF domain in solution is critical for its solubility. The structure of homo-trimeric coiled-coil domain of TRAF2 and hetero-trimeric structure of TRAF1 and TARF2 complex, which is composed of two TRAF2 coiled-coil and one TARF1 coiled-coil domains, were solved^26^. This structure contains coiled-coil domain of TRAF1 starting residue from 266 to 328, while our structure contains coiled-coil domain of TRAF1 starting residue from residue 227 to 261.

### Structural comparison with other TRAF domains

**Figure 4:**
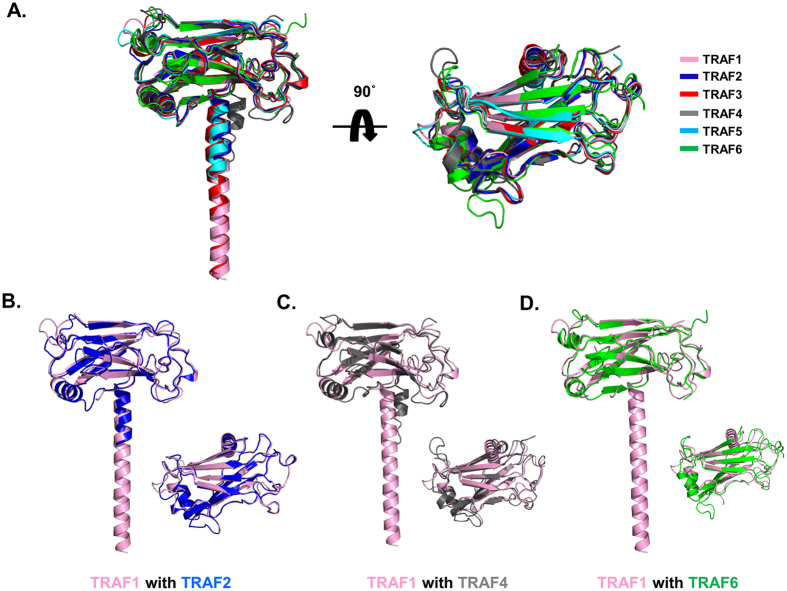
Superposition of the TRAF1 TRAF domain with other TRAF family members. (**A**) TRAF1 TRAF domains are superimposed with five structural homologues. (**B**–**D**). Pairwise structural comparisons. TRAF1 is colored pink and each counterpart is colored blue for TRAF2 (**B**), grey for TRAF4 (**C**) and green for TRAF6 (**D**). Side and top views are shown on the left and right side, respectively.

To compare the TRAF1 structure with other similar structures, the current TRAF1 structure was deposited in the DALI server^27^ and structurally related proteins were identified (Table 2). The top six matches, which had Z-scores of 22.8 to 15.4, were TRAF2^28^, TRAF3^25^, TRAF5^25^, TRAF6^29^, TRAF4^30^, and MEPRIN 31in order, indicating that TRAF2 is the most structurally similar TRAF (Fig. 4A,B). TRAF1 plays a role in regulation of cell survival and apoptosis via formation of homotrimer or heterotrimer with TRAF2, making E3 ubiquitin-protein ligase complex that promotes ubiquitination of target proteins, such as MAP3K14; therefore, the structural similarity between TRAF1 and TRAF2 is understandable. Structurally similar TRAF1 can form a trimeric complex with TRAF2, recruiting the anti-apoptotic E3 ubiquitin ligases BIRC2 and BIRC3 to target receptors. Pair-wise structural alignments between the TRAF1 TRAF domain and other TRAF domains showed that the position and length of several loops in the TRAF1 TRAF domain differed from those of other TRAF domains (Fig. 4A–D). The location of the α_n_ helix of the coiled-coil domain also differed in position. Especially, the α_n_ helix of the coiled-coil domain of TRAF4 is out-layered when compared with the αn helix of the coiled-coil domain of TRAF1 (Fig. 4C). TRAF4 and TRAF6 contains a longer β5–β6 loop and α1–β2 loop, respectively, than those of TRAF1 (Fig. 4C,D). These structural differences of TRAF1 might be critical to its functional differences from other TRAFs. Our current structure of TRAF1 showed the longest TRAF-N coiled-coil domain.

**Figure 5:**
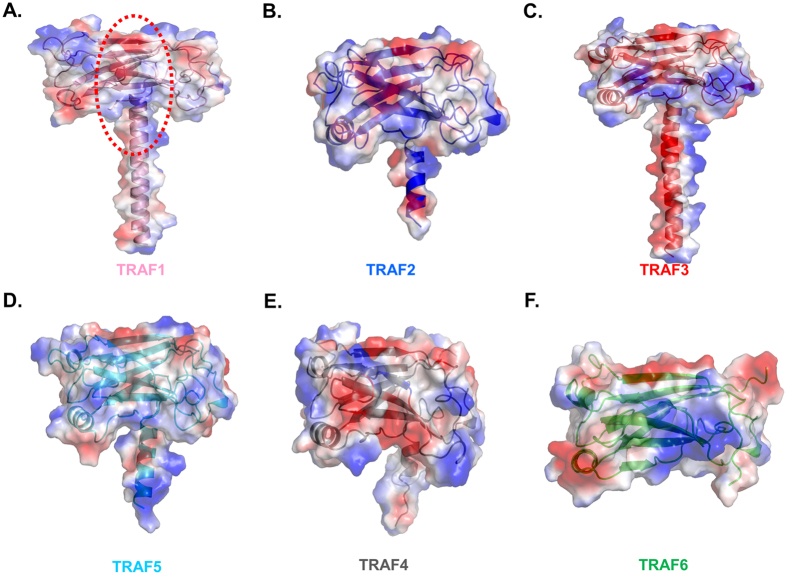
Electrostatic surface representation of the TRAF1 TRAF domain and its structural homologues. (**A**) Electrostatic surface representation of the TRAF1 TRAF domain receptor binding pocket for the TRAF family is indicated by the red-dot circle. (**B**–**F**) Electrostatic surface representation of TRAF 2(**B**), TRAF3 (**C**), TRAF4 (**D**), TRAF5 (**E**) and TRAF6 (**F**) generated by COOT.

Biochemical and structural analyses have led to identification of many interaction hot spots on the TRAF domain with many receptors. Those studies indicated that β3, β4, β6, and β7 of the TRAF domains participate in the receptor interaction (Fig. 5A). Since the surface features often provide their mode of interactions with partners, the electrostatic surface of the TRAF1 TRAF domain was calculated and compared with those of other TRAF families. The TRAF domain of TRAF1 has similar gross features in its electrostatic surface, with TRAF2, TRAF3, and TRAF5 in that the surface is composed of mixed positive and negative charges with several uncharged regions (Fig. 5B–D). TRAF4 contains a more negatively charged surface in the middle of the binding interface (Fig. 5E), while TRAF6 contains a more positively charged surface (Fig. 5F). Because the TRAFs diversely charged surface has been shown to be important to accommodating many diverse receptors in the same binding pocket, TRAF1 might interact with diverse receptors in a method similar to that used by TRAF2 and TRAF3. The presence of similar features of binding surfaces with functionally similar TRAFs, TRAF2 and TRAF3, and different features of binding surface with functionally different TRAF, TRAF4 and TRAF6 may indicate that the surface features dictate the mode of interaction between TRAF1 and its partners.

### Model of TRAF1 interactions with its various receptors

**Figure 6:**
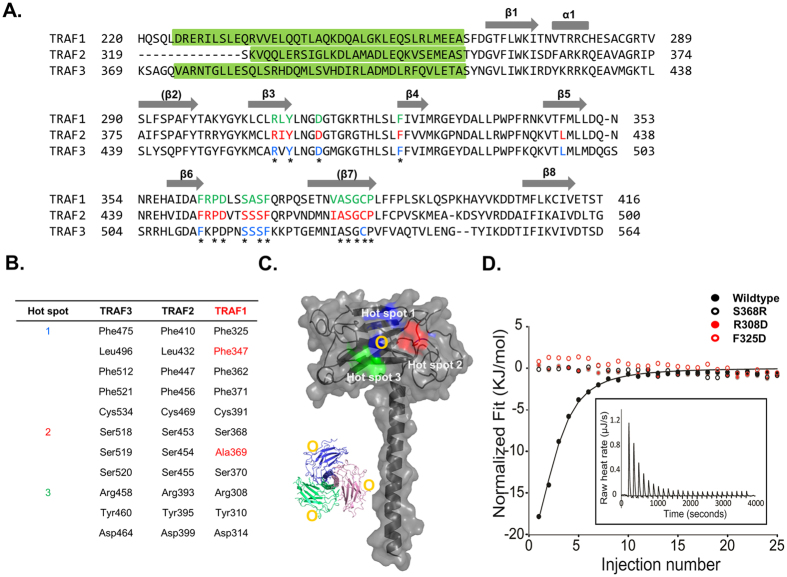
Mapping of proposed binding hot spots onto the TRAF1 TRAF domain. (**A**) Structural based sequence alignment. The surface residues involved in the interaction with binding receptors on TRAF2 and TRAF3 are shown in red and blue, respectively. Conserved residues on the binding hot spots of other TRAF family members including TRAF2 and TRAF3 are shown in green. Completely conserved residues are indicated by stars. Green highlight box indicates TRAF-N coiled-coil domain. (**B**) Conserved residues on the binding hot spots of TRAF2 and TRAF3. Residues not conserved on TRAF1 are indicated by red. (**C**) Conserved exposed residues that might be involved in the receptor interaction are mapped onto the TRAF1 TRAF domain. The three expected binding hot spots on the TRAF1 TRAF domain are indicated by blue (Hot spot 1), red (Hot spot 2), and green (Hot spot 3). Yellow circles indicate the position on the trimeric structure shown on the left side panel. (**D**) Isothermal titration calorimetric analysis of the binding interaction of TANK peptide to TRAF1 wildtype and three mutants (Black closed circle: wildtype, Red closed circle: R308D mutant, Red open circle: F325D, Black open circle: S368D mutant), showing that only wildtype TRAF1 interacts to TANK peptide. Integrated isotherm of the titration and experimental fit to a single site model. A total of 25 injections were performed to measure the interactions.

TRAFs are important adaptor molecules that mediate cellular signaling events. These compounds can interact with various receptors and intra-cellular proteins including CD40, TRADD, LMP1, TNFR2, RANK, and TANK during the particular signaling events. Structural and biochemical studies of TRAFs and their interaction proteins showed that TRAFs use three regions (called binding hot spots), β3 and the loop connecting β3 and β4 (hot spot 3, also called the polar pocket), β6 and the loop connecting β6 and β7 (hot spot 2, also called the serine finger), and many dispersed areas including β4, β5, β6 and β7 (hot spot 1, also called the hydrophobic pocket), to accommodate their various partner molecules^28^,^29^,^32^,^33^. The typical binding hot spots of TRAFs have been well-studied in TRAF2 and TRAF3. The minimal consensus motif in TRAF binding proteins, including TNF-R family members, for TRAF2 and TRAF3 interaction is Px(Q or E)E, although the presence in position 5 of the acid or polar amino acids is favored. In the case of TRAF6, the mode of interaction was unique among TRAF family members. The TRAF6 consensus motif is six amino acids long and the sequence is PxExx (acid or aromatic). To identify the residues that might be involved in protein interactions for signaling events, the TRAF1 sequence was aligned with that of TRAF2 and TRAF3, which have high sequence identity (57% and 52%, respectively) (Fig. 6A). Surface residues that have participated in the receptor interactions with TRAF2 and TRAF3 were marked on the sequences (Fig. 6A). Among those residues involved in the three interaction regions, conserved residues, which might also be involved in the interaction of TRAF1 with its receptor, were marked on the sequence of TRAF1 and mapped on the surface of the TRAF1 TRAF domain (Fig. 6A–C). Most of the residues on the surface of TRAF1 that might be involved in the receptor interaction, including Phe325, Phe462, Phe371, Cys391, Ser368, Ser370, Arg308, Tyr310, and Asp314, are conserved among TRAF domains (Fig. 6A,B). Mapping analysis suggests that this extensive surface of the TRAF1 TRAF domain, which contains both hydrophobic and charged residues, may be involved in interactions with various receptors. To prove our hypothesis, we analysed the interactions between TRAF1 and TANK peptide. TANK, also called I-TRAF, is a modulator of TRAF signalling serving as either an activator or an inhibitor^34^,^35^. TANK performed its function by binding with several TRAF proteins including TRAF2 and TRAF3 as well as TRAF1^34^. TANK peptide (SVPIQCTDKT) is well-known binding part of TANK to TRAFs^36^. TANK peptide interaction to TARF2 and TRAF3 is already reported with various interaction study^36^. The binding ability and affinity of the TANK peptide for TRAF1 was analysed by isothermal titration calorimetry (ITC). The titration of TRAF1 with TANK peptide is shown in Fig. 6D. The interaction exhibits a dissociation constant (K_D_) of 13.6 uM. This affinity of TANK peptide is somewhat higher than affinity reported for TRAF3 interaction (K_D_ of 23.9 uM), indicating that TANK can interact to TRAF1 more tightly. Finally, we produced F325D, S368R, and R308D mutations to analyse the interaction sites on TRAF1 and performed ITC. Figure 6D indicates that all three mutations, that disrupt three known hot spots, diminished the interaction of TANK peptide with TRAF1, indicating that TRAF1 interacts to TANK by using all three hot spots. Because TRAF2 and TRAF3 also use three known hot spot to accommodate TANK, we conclude thatTRAF1 interacts to its binding partners in the similar manner that is used by TRAF2 and TRAF3 for TRAF1-mediated signaling events. Further structural studies, especially of the complex between TRAF1 and its binding receptor, are needed to elucidate the TRAF1-mediated signaling event. In conclusion, the structure of the TRAF1 TRAF domain presented here provides a first step toward elucidation of the molecular basis of the TRAF1 signaling pathway.

## Methods

### Protein expression and purification

The expression and purification methods used in this study have been described elsewhere in detail. Briefly, human TRAF1 TRAF domain corresponding to amino acids 220–416 was cloned into pET24a vector and expressed in *E. coli* BL21 (DE 3) by overnight induction at 20 °C. The protein contained a carboxyl terminal His-tag and was purified by nickel affinity and gel filtration chromatography. A superdex 200 gel filtration column 10/30 (GE healthcare) that had been pre-equilibrated with a solution of 20 mM sodium citrate at pH 5.0 and 1 M NaCl was used for gel-filtration chromatography. The protein eluted at around 16 ml upon gel-filtration chromatography was collected and concentrated to 5–6 mg/ml for crystallization.

### MALS

The molar mass of the TRAF1 TRAF domain was determined by multi angle light scattering (MALS). The target protein was loaded onto a Superdex 200 HR 10/30 gel-filtration column (GE Healthcare) that had been pre-equilibrated in buffer containing 20 mM sodium citrate at pH 5.0 and 1 M NaCl. The acta chromatography system was coupled to a MALS detector (mini-DAWM treos) and a refractive index detector (Optilab DSP) (Wyatt Technology).

### Crystallization and data collection

Crystallization was conducted at 20 °C by the hanging drop vapor-diffusion method using various screening kits. The crystals used for the x-ray diffraction study were grown on plates by equilibrating a mixture containing 1 μl of protein solution (5–6 mg ml-1 protein in 20 mM sodium citrate at pH 5.0 and 1 M NaCl) and 1 μl of a reservoir solution containing 1.26 M ammonium sulfate, 0.1 M CHES pH 9.5, and 200 mM NaCl against 0.3 ml of reservoir solution. A 2.8 Å native dataset was collected at the BL-4A beamline of the Pohang Accelerator Laboratory (PAL), Republic of Korea. Data processing and scaling was carried out using HKL2000^37^.

### Structure determination and analysis

The structure was determined by the molecular replacement phasing method using Phaser^38^. The previously solved TRAF3 structure (PDB code: 4GHU)^25^, which was 49% homologous with TRAF1, was used as a search model. Model building and refinement were performed in COOT^39^ and Refmac5^40^, respectively. Water molecules were added automatically with the ARP/wARP function in Refmac5, then examined manually for reasonable hydrogen bonding possibilities^41^. The quality of the model was checked using PROCHECK and found to be reasonable. A total of 94.6% of the residues were shown to be located in the most favorable region, while 5.4% were in the allowed regions of the Ramachandran plot. The data collection and refinement statistics are summarized in Table 1. Ribbon diagrams and molecular surface representations were generated using the Pymol program^42^.

### Solubility assay

The general strategy of the solubility assay was based on the method introduced by Bondos and Bicknell^43^. Briefly, purified TRAF domain and TRAF-N domain from gel-filtration chromatography in 20 mM sodium citrate and 1 M NaCl buffer were incubated for various times, as indicated. A total of 300 μl of the 400 μl solution of each sample was used for the turbidity assay. The turbidity of each sample was measured directly by the optical density at 600 nm using a spectrophotometer.

### Sequence alignment

The amino acid sequence of TRAFs was analyzed using Clustal Omega (http://www.ebi.ac.uk/Tools/msa/clustalo/).

### Mutagenesis

Site-directed mutagenesis was performed using the Quickchange kit (Stratagene) following the manufacturer’s protocols. Mutagenesis was then confirmed by sequencing. Mutant proteins were prepared using the same method described above.

### Isothemal titration calorimetry (ITC)

Isothermal titration calorimetry experiments were performed by NanoITC (TA Instruments). The wildtype and mutants proteins were dialyzed intensively against PBS buffer, and the TANK peptide (SVPIQCTDKT) was dissolved in the same buffer to minimize heats of dilution. TANK peptide was synthesized and purified by Peptron (Dae-jeon, South Korea). Prior to titration, the protein samples and the peptide were contrifugated at 13,000 rpm at 4 °C for 5 min to remove any debris. For each titration, a concentrated peptide solution (1 mM) was injected into a cell containing wildtype or each mutant of TRAF1 at a concentration of ~20 uM. All the titrations were carried out 15 °C with 25 injections at 160 sec intervals. Binding isotherms were analysed by using the software provided by TA instruments. Baseline controls were acquired with buffer and pure peptide solutions.

## Additional Information

**Data availability**: Coordinates and structural factors were deposited in the Protein Data Bank under PDB ID code 5E1T.

**How to cite this article**: Kim, C. M. *et al.* Crystal structure of TRAF1 TRAF domain and its implications in the TRAF1-mediated intracellular signaling pathway. *Sci. Rep.* **6**, 25526; doi: 10.1038/srep25526 (2016).

## Figures and Tables

**Table 1:** Crystallographic statistics.

Data collection	Native
X-ray source	Synchrotron (PAL 5C)
Detector	ADSC Quantum 315r
Wavelength	0.97760
Space group	P2_1_2_1_2_1_
Cell dimensions	
*a*, *b*, *c*	75.3 Å, 79.4 Å, 108.0 Å
Resolution	50–2.8 Å
Wilson B-factor	58.57 Å^2^
†No.of unique reflections overall	15,860 (729)
†*R*_sym_	6% (19%)
†*I*/σ*I*	31.2 (4.4)
†Completeness	94.7% (91.8%)
†Redundancy	5.0 (3.3)
Refinement	
Resolution	30–2.8 Å
No. of reflections used (completeness)	15,792 (94.8%)
No. of non-H protein atoms	4,599
No. of water molecules	13
No. of ions	0
†*R*_work_	21.0% (26.8%)
†*R*_free_	26.2% (29.1%)
Average B-factors	
Protein	52.1 Å^2^
Water and other small molecules	42.4 Å^2^
r.m.s. deviations	
B-factor for bonded atoms	8.86
Bond lengths	0.011 Å
Bond angles	1.442°
MolProbity analysis	
Ramachandran outliers	0.00%
Ramachandran favoured	95.35%
Ramachandran allowed	4.65%
Rotamer outliers	0.39%
C-beta deviations	0
Clashscore	16.41

^†^Highest resolution shell is shown in parenthesis.

**Table 2:** Structural similarity search using DALI^27^.

Proteins and accession numbers	Z-score	RMSD (Å)	Identity (%)	References
TRAF2 (1CA9)	22.8	1.2	57	28
TRAF3 (4GHU)	21.8	1.2	52	25
TRAF5 (4GJH)	21.2	1.2	49	25
TRAF6 (1LB4)	19.1	1.4	40	29
TRAF4 (4K8U)	18.1	2.2	40	30
MEPRIN (4GWN)	15.4	7.631	26	31
